# A MicroRNA-Transcription Factor Blueprint for Early Atrial Arrhythmogenic Remodeling

**DOI:** 10.1155/2015/263151

**Published:** 2015-06-28

**Authors:** Mario Torrado, Diego Franco, Estefanía Lozano-Velasco, Francisco Hernández-Torres, Ramón Calviño, Guillermo Aldama, Alberto Centeno, Alfonso Castro-Beiras, Alexander Mikhailov

**Affiliations:** ^1^Institute of Health Sciences, University of La Coruña, 15006 La Coruña, Spain; ^2^Department of Experimental Biology, University of Jaén, Jaén, Spain; ^3^University Hospital Center of La Coruña, La Coruña, Spain

## Abstract

Spontaneous self-terminating atrial fibrillation (AF) is one of the most common heart rhythm disorders, yet the regulatory molecular mechanisms underlying this syndrome are rather unclear. MicroRNA (miRNA) transcriptome and expression of candidate transcription factors (TFs) with potential roles in arrhythmogenesis, such as *Pitx2*, *Tbx5*, and myocardin (*Myocd*), were analyzed by microarray, qRT-PCR, and Western blotting in left atrial (LA) samples from pigs with transitory AF established by right atrial tachypacing. Induced ectopic tachyarrhythmia caused rapid and substantial miRNA remodeling associated with a marked downregulation of *Pitx2*, *Tbx5*, and *Myocd* expression in atrial myocardium. The downregulation of *Pitx2*, *Tbx5*, and *Myocd* was inversely correlated with upregulation of the corresponding targeting miRNAs (miR-21, miR-10a/10b, and miR-1, resp.) in the LA of paced animals. Through *in vitro* transient transfections of HL-1 atrial myocytes, we further showed that upregulation of miR-21 did result in downregulation of *Pitx2* in cardiomyocyte background. The results suggest that immediate-early miRNA remodeling coupled with deregulation of TF expression underlies the onset of AF.

## 1. Introduction

Atrial fibrillation (AF), a heart rhythm disorder dubbed as “the epidemic of the 21st century” [1], represents nowadays a serious clinical problem making the development of novel treatment approaches highly desirable (recently reviewed in [2]). AF is generally considered to be a progressive condition, occurring first in a paroxysmal form (short-lived self-terminating episodes) and then in persistent and eventually long-lasting permanent forms. Recent growing evidence indicates that the underlying molecular mechanisms are distinct in each form of AF [3, 4]. Proarrhythmogenic molecular remodeling, broadly defined as any change in atrial gene regulation that promotes atrial conduction disturbances, is potentially crucial for unraveling the onset mechanisms of atrial tachyarrhythmias.

There is emerging evidence that microRNAs (miRNAs) can modulate a diverse spectrum of cardiac functions through their ability to remodel cardiac transcriptional circuits (reviewed in [5–7]). The tight regulation of the levels of miRNAs is critical for maintaining normal physiological conditions, and dysregulated miRNA levels contribute to the development of heart diseases (reviewed in [8–10]). In relevance to this study, miRNAs have been demonstrated to be essential in regulating atrial excitability and can be involved, directly or indirectly, in increased atrial arrhythmogenicity and AF (reviewed in [11–15]). In this context, there is growing evidence regarding the aberrant miRNA expression in chronic AF conditions in patients [16–22], while only a few papers have focused on miRNA transcriptomic profiling of atrial samples from patients with paroxysmal AF [23, 24]. Specifically, these papers demonstrated that a miRNA-mediated imbalance in the gonadotropin releasing hormone receptor/p53 [23] and metalloproteinase/inhibitor [24] pathways may potentially be involved in atrial remodeling. As it is mentioned in the reports, fibrillating atrial samples were collected during cardiac surgery in hypertonic patients [23] or in patients with long-lasting (years) paroxysmal AF [24]. Each of these clinical conditions is known to be associated with a chronic atrial stretch and consequent atrial structural remodeling. In this sense, left atrial enlargement was observed in patients in the study by [24].

Although these studies provide evidence in support of the importance of miRNAs in long-lasting AF, the use of miRNA expression profiling as a tool to assess the very-early atrial remodeling in animal models of self-terminating atrial tachyarrhythmias (resembling early-onset lone paroxysmal AF in humans) is still missing. It is important to highlight that the left (LA) and right (RA) atria have different susceptibilities towards developing arrhythmias, given the fact that the pulmonary veins and surrounding myocardial regions of the LA constitute the most frequent foci of AF initiation.

In the current study, we report the changes in miRNA expression in the LA in response to ectopic tachyarrhythmia episodes induced by short-term atrial pacing in pigs. 38 microRNAs were differentially expressed, 18 of which were upregulated in the LA of paced versus sham (control) animals. In addition, we identified expression differences for novel miRNAs that have not previously been annotated as either cardiac- or AF-associated.

Alterations in atrial miRNAs can contribute to transcription factor (TF) deregulations underlying early atrial remodeling. Genome-wide association studies and other approaches have identified the TFs,* Pitx2* and* Tbx5* as candidate genes linked to susceptibility for early development of AF (reviewed in [41, 42]) and myocardin (*Myocd*) as a transcription cofactor linked with LA functional and structural remodeling [43]. Interestingly,* Pitx2*,* Tbx5*, and* Myocd* expression is markedly downregulated in the LA in response to ectopic tachyarrhythmia. Particularly intriguing is that the downregulation of these TFs inversely correlates with upregulation of the corresponding targeting miRNAs in the LA of paced animals.

## 2. Materials and Methods

### 2.1. Animals and Experimental Design

**Figure 1:**
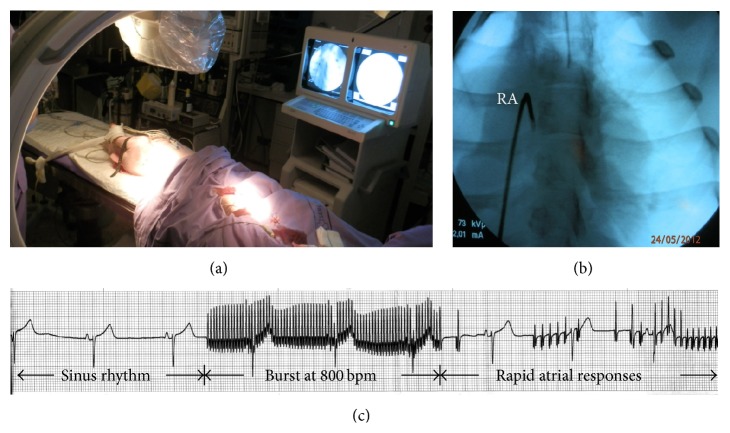
An electrical pacing assay. (a) Anaesthetized piglet (with automatic artificial lung ventilation) in the animal operating room. (b) Placing a pacing catheter in the right atrium (RA) using fluoroscopic guidance. (c) Representative telemetry ECG recordings monitored before, during, and after RA burst pacing.

“Large white” 3-month-old pigs were obtained from a local commercial breeder (La Coruña, Spain) and randomly divided into two groups: (1) sham (*n* = 5; mean body weight, 25.2 ± 1.2 kg) and (2) atrial tachypacing (*n* = 5; mean body weight, 25.7 ± 0.8 kg). A close-chest tachyarrhythmia model was established in animals via rapid atrial electrical stimulation with a controlled ventricular response rate. To control anaesthesia, ventilation, and oxygenation, ECG, heart rhythm, and blood pressure were continuously monitored. The right femoral vein was dissected and cannulated with a multielectrode catheter (Medtronic, Minneapolis, USA) connected with an external pulse stimulator (A-M Systems, UK) for programmed pacing rates. Under fluoroscopic guidance, the catheter was passed into the right atrium (Figure 1). To avoid tachycardia-induced ventricular dysfunction (ventricular arrhythmias), esmolol hydrochloride (a beta adrenergic receptor blocker) was given before pacing. After a 5-minute stabilization of sinus rate, a three-burst-pacing (at 800 bpm) protocol was performed in each animal of the paced group. Occurrence and duration of rhythm disturbances were characterized by ECG (Figure 1). The duration of induced self-terminating paroxysms of AF was measured from the end of the last stimulus of the burst pacing to the first P-wave upon spontaneous reversion to normal sinus rhythm. Burst pacing was not performed in the sham-operated group. Animals were euthanized 20–24 hours after cessation of pacing to harvest cardiac tissues for RNA and protein isolation and histology. Animals were used in accordance with the European Commission Directive 86/609/EEC and all protocols were approved by the Institutional Animal Care and Use Ethics Committee (permit number: CE 012/2012; University of La Coruña, La Coruña, Spain).

### 2.2. Cell Culture and Transfection In Vitro

The mouse atrial cardiomyocyte cell line, HL-1 (provided by Professor William C. Claycomb), was cultured in Claycomb's growth medium. HL-1 cells (6 × 10^5^ cells per well) were transfected with pre-miR-21 (Ambion, USA) and anti-miR-21 (Eurogentec, Belgium) at 10–50 nM using Lipofectamine 2000 (Invitrogen, Barcelona, Spain) according to manufacturer's guidelines. Negative controls included nontransfected (mock) cells as well as 5′ carboxyfluorescein- (FAM-) labeled pre-miR negative control transfected cells, which also allowed transfection efficiency evaluation. In each assay, transfections were performed in triplicate. The transfection efficiency was around 60% in all experiments. After 4 hours of posttransfection, HL-1 cells were cultured in fresh medium for 48 hours and then harvested and processed for RNA and protein extraction as described [44].

### 2.3. Microarray

Purification of total RNA, including small RNAs, from LA samples was performed using miRNeasy Mini Kit (Qiagen, USA), including the on-column DNase digestion with RNase-free DNase, as described in the Qiagen Manual. Deep-frozen LA samples were directly homogenized in QIAzol lysis reagent (Qiagen) using a high-speed rotor-stator homogenizer (Ultra-Turrax T8, Germany). RNA was quantified by spectrophotometry and quality was evaluated by gel electrophoresis. Microarray analysis of RNAs isolated from LA biopsies of three paced and three sham pigs was performed by a service provider (LC Sciences, Houston, USA) using Pig miRNA Array (MRA-1013, version miRPig_20), which contains 322 unique probes (9 repeats each) of pig mature miRNAs. The probe content comes from version 20 of the miRBase sequence database which was updated on June 24, 2013. Briefly, the detection probes were made by* in situ* synthesis using PGR (photogenerated reagent) chemistry. Hybridization was performed overnight on a *μ*Paraflo microfluidic chip using a microcirculation pump (Atactic Technologies). Fluorescence images were collected using a laser scanner (GenePix 4000B, Molecular Device) and digitized using Array-Pro image analysis software (Media Cybernetics). *t*-test was performed between control and experimental sample groups. *T*-values were calculated for each miRNA, and *p* values were computed from the theoretical *t*-distribution. The clustering was done using the hierarchical method and performed with average linkage and Euclidean distance metric. The complete microarray data is available at NCBI through GEO (Gene Expression Omnibus) accession number GSE65330 (http://www.ncbi.nlm.nih.gov/geo).

### 2.4. Real-Time Quantitative PCR (qRT-PCR)

qRT-PCR was performed on Bio-Rad IQ5 instrument (Bio-Rad, Madrid, Spain) and MxPro Mx3005p qPCR thermal cycler (Stratagene, Madrid, Spain) using, respectively, SYBR Green (Bio-Rad, Madrid, Spain) and Dynamo SYBR Green (Finnzymes, Finland) master mix as described previously [44, 45]. The primer pairs were located in different exons to rule out genomic DNA amplification. Each primer pair used yielded a single peak of dissociation on the melting curve and a single band with the expected size on PAGE gels. Identity of the PCR products was confirmed by sequencing. NT and non-RT RNA template reactions were used as negative controls. All PCR setups were performed at least in triplicate. Relative quantifications were calculated with the comparative ΔCt cycle method with normalization to the expression of housekeeping genes coding for ribosomal protein L19 (*Rpl19*), *β*-actin, glyceraldehyde-3-phosphate dehydrogenase (*Gapdh*), and *β*-*D*-glucuronidase (*Gusb*). The efficiency of target and reference amplifications was tested to be approximately equal. miRNA qRT-PCR was performed using Exiqon LNA microRNA qRT-PCR primers and detection kit (Exiqon, Madrid, Spain) according to manufacturer's guidelines. All reactions were run in triplicate using 5S as normalizing control. Primer sequences and additional data are available upon request.

### 2.5. Antibodies

The following primary antibodies were used: (1) rabbit polyclonal antibodies to PITX2A,B,C (Capra Science, Ängelholm, Sweden; at 1 : 2000 dilution): the specificity of the anti-PITX2 antibodies was independently validated by Western blot analysis of COS-7 cells expressing each PITX2 isoform [46]; (2) rabbit polyclonal antibodies to TBX5 (Abcam, Cambridge, UK; at 1 : 500 dilution); (3) rabbit polyclonal antibodies to myocardin which were generated by Davids Biotechnologie (Regensburg, Germany) using the recombinant TAD-containing fragment of porcine MYOCD as immunogen (at 1 : 500 dilution): these antibodies were shown to be specific for both MYOCD-A (minor) and MYOCD-B (major) variants expressed in pig cardiac tissues [47]; (4) rabbit polyclonal antibodies to cardiac troponin I (Abcam, Cambridge, UK; at 1 : 40000 dilution); (5) rabbit polyclonal antibodies to cardiac calsequestrin-2 (Abcam, Cambridge, UK; at 1 : 10000 dilution); (6) rabbit monoclonal antibodies to sarcoplasmic reticulum ATPase (SERCA-2A; Abcam, Cambridge, UK; at 1 : 10000 dilution); (7) rabbit polyclonal antibodies to caspase-3 (Cell Signaling, Leiden, Netherlands; at 1 : 1000); (8) rabbit monoclonal antibodies to caspase-9 (Abcam, Cambridge, UK; at 1 : 1000 dilution); and (9) mouse monoclonal anti-GAPDH (glyceraldehyde-3-phosphate dehydrogenase) antibody (Sigma, Madrid, Spain; at 1 : 10000 dilution). Secondary peroxidase conjugated anti-rabbit and anti-mouse IgG (Fab-specific) antibodies were purchased from Sigma (Madrid, Spain).

### 2.6. SDS-PAGE and Western Blotting

Tissue samples were homogenized in standard 2x Laemmli buffer (Invitrogen, Barcelona, Spain) supplemented with complete protease inhibitor cocktail (Roche, Madrid, Spain) as previously described [45, 46]. Following centrifugation at 20000 g for 30 minutes, the concentration of supernatant proteins was analyzed using the Bio-Rad DC Protein Assay Kit (Bio-Rad, Hercules, USA) according to the manufacturer's protocol. The protein extracts were normalized to total protein concentration; the results of normalization were confirmed by SDS-PAGE and Coomassie staining before Western blotting analysis [46]. Protein supernatants (loading range of 5–15 mg/run) were resolved on a 12% SDS-PAGE (Mini-Protean-III, Bio-Rad, Hercules, USA) and blotted onto PVDF-membranes (Hybond-P, Amersham Biosciences, Barcelona, Spain). Molecular weight (MW) standards (Precision Plus Protein WesternC Standards from Bio-Rad and SeeBlue Plus2 Pre-Stained Standard from Invitrogen) were included on each gel. Blots were probed with the antibodies indicated above and visualized by the Super-Signal West Pico chemiluminescent substrate (Pierce Biotechnology, Madrid, Spain). Equivalence of protein loading was confirmed by Amido Black 10B (Merck, Barcelona, Spain) staining of blots after immunodetection. The blots were reprobed with anti-GAPDH antibodies as additional control for loading. Quantification of Western blot signals was obtained by using a Bio-Rad GS800 calibrated densitometer with Quantity One software.

### 2.7. Histochemical Staining

Fragments of LA samples were fixed with 4% paraformaldehyde (in PBS, 1 hour at room temperature), paraffin-embedded, 5 *μ*m sectioned, stained with Picrosirius Red (Sigma, Madrid, Spain) as described previously [48], and visualized with bright-field “Nikon Eclipse 600” microscopy by an experienced technician blinded to the experimental design. The area of red-stained pixels was quantified in three zones of LA free wall sections from three paced versus three sham animals by ROI (Region of Interest) analysis using ACT-2U Image software (version 1.4, Nikon, Japan).

### 2.8. Statistical Analysis

Values are expressed as mean ± S.E.M. All comparisons between groups were performed using unpaired Student's *t*-test. Differences were considered statistically significant for *p* value ≤ 0.05.

## 3. Results

### 3.1. Burst Tachypacing Results in Self-Terminating Paroxysms of Atrial Fibrillation

Brief self-terminating episodes of AF were induced in postnatal pigs by repetitive burst pacing of the RA via an implanted catheter electrode (see Figure 1). Hemodynamic parameters were comparable in the paced versus sham animals throughout the experiment. Before and after catheter insertion, the ventricular rate means were similar at baseline in both sham and pacing groups but increased in the pacing group compared with baseline upon electrical stimulation (150 ± 6 bpm versus 130 ± 3 bpm). Every burst was followed by consecutive (3–5) AF episodes, each lasting 20–30 sec. On average, a total duration of AF episodes (per animal) was 600 ± 120 sec. No AF episodes were detected by ECG-tracing at least 1 hour after the tachyarrhythmia protocol was terminated. On the next day, the ECG examination (prior to sacrifice of animals and excision of the heart) showed normal heart rhythm (with normal QRS duration) in both paced and sham pigs. Picrosirius red staining revealed no signs of fibrosis in LA samples from paced or sham animals (data not shown).

These results suggest that episodes of self-terminating AF observed in our porcine model are reminiscent of those which occur in patients with asymptomatic paroxysmal AF.

### 3.2. Paroxysmal Atrial Tachyarrhythmia Leads to a Rapid Change in Atrial miRNA Transcriptome

**Figure 2:**
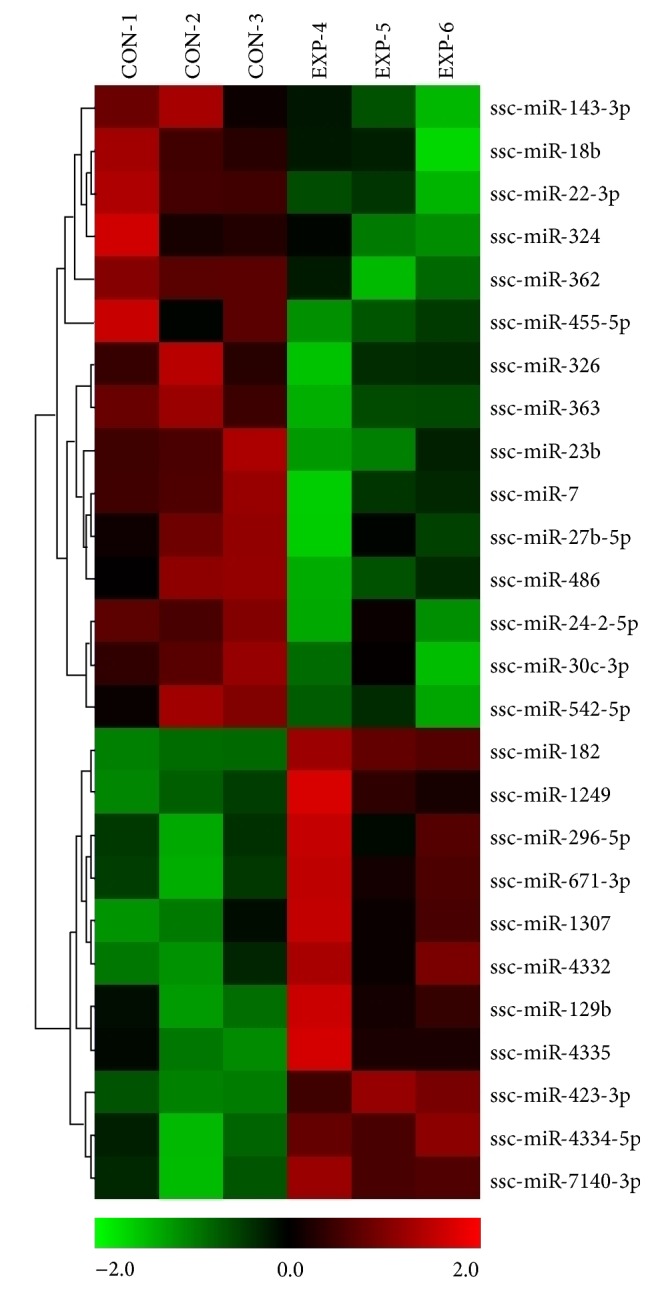
MicroRNA microarray expression profiling of the left atrium from paced versus nonpaced pigs. Heat map of the most differentially expressed (statistically significant) miRNAs in the left atrium of three paced (4–6) compared to three sham (1–3) animals. Relative expression is log_2_.

Since the RA might have been damaged by catheter insertion, we studied miRNA expression changes associated with AF in the LA from paced versus sham pigs 20–24 hours after pacing. Our initial goal was to analyze AF-induced miRNA transcriptome changes by microarray approach. The results of microarray experiments demonstrated that transient episodes of AF alter the miRNA expression profiles of the LA. Of 322 porcine mature miRNA sequences analyzed by microarrays, the expression of 29 miRNAs was significantly up- (12 miRNAs) or downregulated (17 miRNAs) in the LA from paced versus sham animals (Figure 2; Table 1). Seven miRNAs (i.e., miR-7140-3p, miR-4335, miR-129b, miR-296-5p, miR-4334-5p, miR-4339, and miR-182) showed 2-fold or greater increases after LA pacing. The biggest difference was observed for miR-7140-3p, which was expressed approximately 4-fold higher in paced than in sham animals. The known cardiac-expressed miRNAs (i.e., miR-24-2-5p, miR-542-5, miR-27b-5p, miR-7, and miR-30c-3p; see Table 1) were prominent among the miRNAs that showed a significant downregulated expression (~2-fold decrease) in the paced group compared with controls. Of particular note, the microarray profiling unveiled a low-to-moderate downregulation of three miRNAs (i.e., miR-143-3p, miR-363, and miR-18b) previously detected in patients with chronic AF. Pacing-induced alterations in the expression of other miRNAs with particular concern for their involvement in AF could not be determined by microarray hybridizations due to a high variability among replicates (miR-1, miR-21, miR-23a/b, miR-29a, and miR-133a) or very low hybridization signals (miR-10a, miR-10b; see the complete microarray data at NCBI through GEO accession number GSE65330).

**Figure 3:**
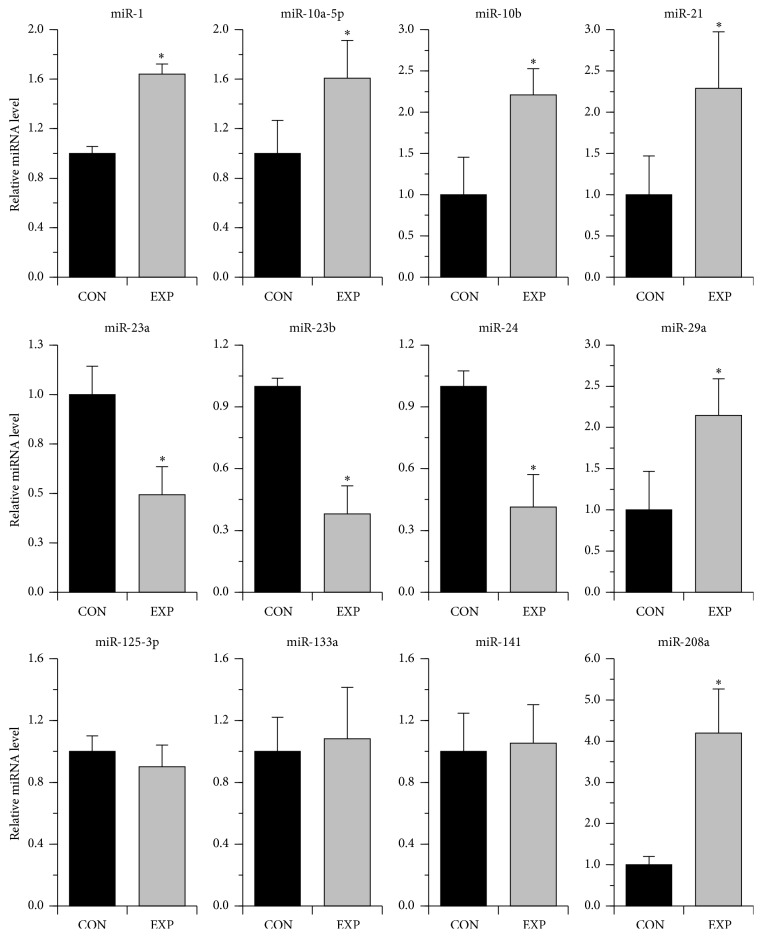
Expression of AF-associated miRNAs in the left atrium from paced (EXP) versus nonpaced (CON) pigs as revealed by qRT-PCR analysis. ^∗^ *p* ≤ 0.05 (*n* = 5 for each group).

Consequently, our second goal was to study whether the expression of these and other AF-associated miRNAs (not revealed by microarray evaluation) could be altered in our porcine model. To this end, the expression of 11 miRNAs, selected on the basis of published reports [7–9, 13, 14, 21] (and references wherein), was analyzed by qPCR in the LA from paced versus sham pigs (Figure 3). Six miRNAs (miR-1, miR-10a-5p, miR-10b, miR-21, miR-29a, and miR-208a) showed a higher expression in paced as compared with nonpaced animals. The expression of miR-23a, miR-23b, and miR-24 was significantly reduced following pacing, while that of miR-133a, miR-125-3p, and miR-141 remained unchanged.

Taken together, the results indicate that short-term atrial tachyarrhythmia episodes lead to a rapid miRNA remodeling of atrial myocardium.

### 3.3. Paroxysmal Atrial Tachyarrhythmia Leads to Downregulation of Transcriptional Factors Related to Atrial Arrhythmia Susceptibility

TFs, such as* Pitx2* and* Tbx5*, are increasingly recognized as potentially important contributors to atrial arrhythmia susceptibility (recently reviewed in [42]). Enlargement of the LA is widely regarded to be an epidemiological risk indicator for AF, and a transcription cofactor, myocardin (*Myocd*), has been identified as a possible susceptibility gene affecting LA size and function [43]. Our objective in this regard was to study whether these genes (with potential roles in arrhythmogenesis) are involved in early response of LA myocardium to induction of paroxysmal-like AF.

**Figure 4:**
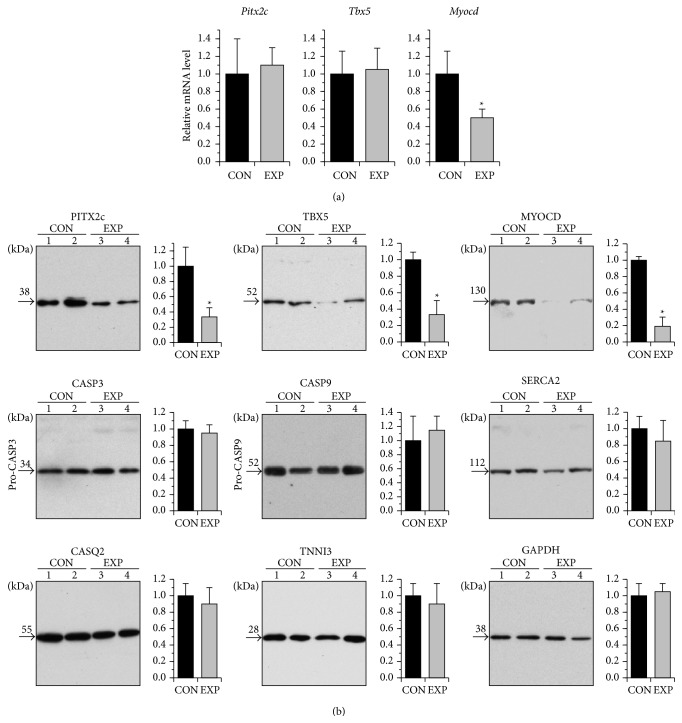
Altered expression of* Pitx2, Tbx5*, and* Myocd* in response to atrial short-term tachyarrhythmia. (a) Average relative values of transcript levels of* Pitx2c*,* Tbx5*, and* Myocd* in the left atrium from paced (EXP) versus sham (CON) animals. ^∗^ *p* ≤ 0.05 (*n* = 5 for each group). (b) Representative Western blots of left atrium samples from sham-operated (CON, lines 1-2) and paced (EXP, lines 3 and 4) animals and overall relative levels of the proteins as based on average values from each group. ^∗^ *p* ≤ 0.05 (*n* = 5 for each group). Western blot replicates were probed with antibodies against PITX2A,B,C, TBX5, MYOCD (myocardin), CASP3 (caspase-3), CASP9 (caspase-9), SERCA2 (cardiac sarcoplasmic reticulum Ca(2+)-ATPase 2a), CASQ2 (cardiac calsequestrin), TNNI3 (cardiac troponin I), and GAPDH (glyceraldehyde-3-phosphate dehydrogenase). MW values of the bands detected are shown.

We evaluated transcript levels of* Pitx2*,* Tbx5*, and* Myocd* in the LA myocardium from paced versus nonpaced control pigs by qRT-PCR (Figure 4(a)). While the level of* Myocd* mRNA expression was reduced following pacing,* Pitx2* and* Tbx5* remained statistically unchanged between control and paced groups. At the protein level, however, a marked decline in expression of not only* Myocd* but also* Pitx2* and* Tbx5* was revealed in the LA of paced animals by Western blot (Figure 4(b)).

Signs of increased apoptosis were detected in atrial tissue in the canine tachypacing model of AF [49]. In this sense, the possibility that apoptosis might be responsible for the observed* Pitx2*,* Tbx5*, and* Myocd* protein downregulation could not be excluded. Western blot analysis did not reveal differences in LA expression levels of pro-caspase-3 and pro-caspase-9 between paced and control pigs, nor signs of activation of these key apoptotic enzymes in any of LA samples studied. In addition, LA levels of cardiac calcium-handling proteins (calsequestrin-2 and sarcoplasmic reticulum Ca^2+^ ATPase) and troponin I were not affected by pacing* per se* (Figure 4(b)). Therefore, it is doubtful that apoptosis is the principal factor responsible for the observed* Pitx2*,* Tbx5*, and* Myocd* protein downregulation in the LA following pacing.

**Figure 5:**
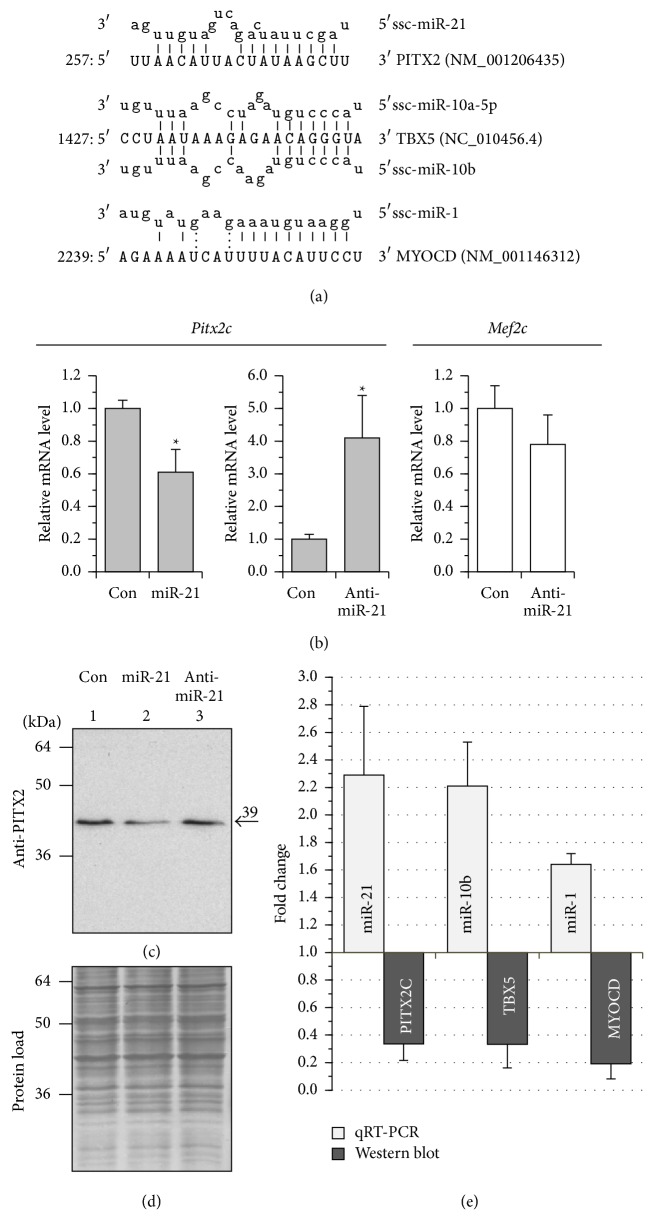
MicroRNAs which can be potentially responsible for the downregulation of* Pitx2*,* Tbx5*, and* Myocd* expression in atrial myocardium after pacing. (a) miRNA:mRNA alignments. Shown are the schematics of porcine* Pitx2* 3′-UTR sequence targeted by miR-21, porcine* Tbx5* 3′-UTR sequence targeted by miR-10a and miR-10b, and human* Myocd* 3′-UTR sequence targeted by miR-1. The porcine* Tbx5* 3′-UTR was identified within the pig genomic chromosome-14 sequence (NC_010456.4), starting from nt 40259322. (b) Overall relative levels of* Pitx2c* (grey boxes) and* Mef2c* (white boxes) transcripts in HL-1 cells transfected with 50 nM miR-21 mimic, anti-miR-21 inhibitor, and FAM-labeled pre-miR negative control (Con). The results of qRT-PCR analysis are shown. Data from 3 replicates of each transfection were pooled and averaged. ^∗^ *p* ≤ 0.05. (c) Control and transfected cells were pooled (from triplicate wells in each setup), lysed, electrophoresed, and immunoblotted with antibodies against PITX2A,B,C. MW values (kDa) of the bands detected are shown. (d) Membrane stained with Amido Black 10B. (e) The histogram demonstrating inverse expression correlations of selected miRNA/target gene pairs in paced pigs as revealed by qRT-PCR (miR-21, miR-10b, and miR-1) and Western blot (PITX2C, TBX5, and MYOCD). See text for details.

It then became our working hypothesis that inhibition of translation by miRNAs can be responsible, at least in part, for the decrease in protein expression of* Pitx2*,* Tbx5*, and* Myocd* in paced pigs. There is growing evidence that miRNAs induce gene silencing with translational repression occurring first and then followed by mRNA decay (reviewed in [50, 51]). Our qPCR analysis showed that pacing resulted in a significant upregulation of a set of AF-associated miRNAs (i.e., miR-1, miR-10a, miR-10b, miR-21, miR-29a, and miR-208a) in the LA compared with the control (see Figure 3). Computational analysis (by miRanda and Targetscan) of the 3′ untranslated region of pig* Pitx2*,* Tbx5*,* and Myocd* genes revealed consensus sites for binding of, respectively, miR-21, miR-10a/miR-10b, and miR-1 (Figure 5(a)). Some of these predicted miRNA/mRNA pairings were in line with the available experimental evidence. In transfection assays, miR-1 inhibits mouse* Myocd* expression at the transcript [52] and protein level [53], while both miR-10a and miR-10b negatively modulate human* Tbx5* expression at the protein level [54].

In this study, we attempted to validate, for the first time, whether miR-21 can alter* Pitx2* expression in a cardiomyocyte background. To this end, miR-21 mimic and anti-miR-21 inhibitor were transfected into atrial HL-1 cardiomyocytes and the expression of* Pitx2c* gene was detected by qRT-PCR and Western blot (Figures 5(b), 5(c), and 5(d)). Transfection of HL-1 cardiomyocytes with miR-21 mimic silenced* Pitx2c *expression to a certain, but statistically significant, extent, whereas anti-miR-21 induced a marked increase in the* Pitx2c* transcript without affecting the expression of* Mef2c* (myocyte enhancer factor 2c) TF. Of note, endogenous miR-21, abundantly expressed in HL-1 cells [55], may interfere with* Pitx2*-silencing by miR-21 mimic transfection. An efficient* Pitx2c* activation by anti-miR-21, as observed in transfection assays, is concordant with this suggestion. A complimentary Western blot analysis (Figures 5(c) and 5(d)) revealed that PITX2C protein levels were significantly decreased in miR-21 transfected cells, being only slightly upregulated after anti-miR-21 delivery. The latter could probably be the result of additional mechanisms influencing the* Pitx2c* transcript versus protein ratio in anti-miR-21 transfected cells.

Consistent with the results obtained and other reports [53, 54], the histogram plot (Figure 5(e)) exhibits a clear inverse correlation in the protein expression of* Pitx2 *versus miR-21,* Tbx5 *versus miR-10b, and* Myocd *versus miR-1 in the LA of paced animals, confirming thus our hypothesis.

Together these data show that sporadic AF paroxysms are associated with marked downregulation of these TFs involved in AF susceptibility of atrial myocardium.

## 4. Discussion

In this study we provide evidence for two major findings. On the one hand, self-terminating paroxysms of AF caused a rapid and substantial miRNA remodeling of the LA myocardium in our animal model. On the other hand, this miRNA remodeling is associated with a marked downregulation of* Pitx2*,* Tbx5*, and* Myocd* TFs in paced atria.

The expression of 38 miRNAs was significantly altered due to burst pacing-induced paroxysms of AF, as revealed by microarray (29 miRNAs, see Table 1) and qPCR analysis (9 miRNAs, see Figure 3), and this number is obviously superior to that found in microarray study of LA samples from patients with paroxysmal AF (10 miRNAs [24]). They can be divided, in terms of expression patterns, into three categories as (1) miRs that have not previously been reported as expressed in the heart, (2) miRs with known expression in cardiac and skeletal muscles but not yet associated with AF, and (3) miRs with well-established AF-associated expression. Although this is by no means an exclusive list, it certainly contains some of the promising miRs to be further evaluated for their potential in AF. Of particular note, in this context, are miR-182 and miR-486, which were found to attenuate atrophy-related gene expression in skeletal muscle [38, 56]. Albeit the assayed tissue was skeletal muscle, both these miRs target the FoxO1 TF which has an important function in mediating apoptosis in the heart [57]. AF is strongly associated with changes in the heart that occur with aging (reviewed in [58]). In this respect, miR-17-3p (see Table 1), a negative modulator of cardiac aging [37], would also be a good candidate to be studied for a potential AF meaning.

The expression signature of known AF-associated miRNAs in our model of paroxysmal-like AF (bold-marked in Table 1 and listed in Figure 3) appears to be different from that found in chronic AF in patients and animal models. Downregulation of miR-1, miR-10a, miR-29a, and miR-208a has been implicated in chronic AF [7, 13, 59], while these miRNAs were highly upregulated in the porcine LA in response to brief paroxysms of AF. In the same way, the levels of miR-133 expression significantly downregulated in chronic AF [13, 14] were unchanged in our model. Upregulation of miR-18b and miR-363 was found to be associated with chronic AF in patients [21], albeit these miRNAs were downregulated in paced pigs (see Table 1). Overall, it is tempting to speculate that the expression of the same set of AF-associated miRNAs can be up- or downregulated as a function of progression from paroxysmal to chronic AF. What is of note in this regard, however, is that the expression of miR-22-3p, miR-23b, and miR-143-3p was similarly downregulated in both patients with chronic AF [20] and our paroxysmal-like AF model (see Table 1). Likewise, expression of miR-21 was upregulated in the LA of patients with long-term persistent AF [60], as well as after short-term AF in pigs (this work). These results suggest that the expression of a subgroup of AF-associated microRNAs could be similarly altered in both paroxysmal and chronic AF states, indicating that congruent miRNA remodeling may occur within different AF contexts.

There are a few data [23, 24] which instead suggest that microRNA remodeling is quite distinct in paroxysmal AF in comparison with that in chronic AF. In one of these reports [23], RA biopsies were obtained from hypertonic patients with paroxysmal AF secondary to or associated with organic heart diseases, suggesting that the observed alterations in miRNA expression may have been attributable to the underlying heart disease rather than paroxysmal AF* per se*. In the other study [24], patients with paroxysmal AF had no obvious clinical signs of cardiovascular disease but LA biopsies taken from them for miRNA microarray analysis showed a markedly increased fibrosis as compared to those from patients with sinus rhythm. Our study is advantageous to the above-mentioned reports in the sense that we performed the miRNA microarray analysis, using LA biopsies from paced animals without pathological cardiovascular background. In addition, biochemical and histochemical analyses did not reveal signs of proapoptotic and profibrotic activation in paced atrial samples.

An aspect of particular interest of our study was the finding that the upregulation of miR-1, miR-10a, miR-10b, and miR-21 is negatively correlated with downregulation of, respectively, MYOCD, TBX5, and PITX2c in the paced LA (see Figure 5(e)). The results of transient transfection assays demonstrated that (1) miR-21 negatively regulates the expression of* Pitx2c* (this work), (2) miR-1 suppresses* Myocd* expression [53], and (3) miR-10a and miR-10b both inhibit* Tbx5* expression [54]. The functional relevance of these miRNA/TF relations (established through our model) to AF development remains elusive and thus future experimental approaches using knockdown and overexpression strategies are highly desirable.

There are lines of evidence that implicate downregulated expression of* Pitx2*,* Tbx5*, and, probably,* Myocd* in a predisposition to AF progression. In fact, several studies revealed an association between LA* Pitx2* downregulation and increased atrial arrhythmogenicity (reviewed in [61]). More recent research implicates* Pitx2* and* Pitx2*-mediated signaling in prevention of AF [62, 63]. In some patients with Holt-Oram syndrome and AF, the disease is caused by* Tbx5* mutations that create a premature termination codon, which is predicted to result in haploinsufficiency. It should be noted, however, that increased* Tbx5* dosage (gain-of-function mutations) was also associated with Holt-Oram syndrome and paroxysmal AF (reviewed in [41, 42]).* Myocd* is regarded as the most likely candidate among the genes affecting LA size [43], which is a clinical marker for risk stratification of AF development [64]. Cardiac-restricted inactivation of the* Myocd* gene in the adult heart resulted in development of severe four-chambered heart failure as a result of massive myocyte loss via apoptosis and replacement fibrosis (reviewed in [65, 66]).

The proarrhythmogenic mechanisms leading to the spontaneously self-terminating AF episodes typical of patients with paroxysmal AF remain elusive and not yet studied as hoped. Our results provide suggestive evidence for a significant* Pitx2*,* Tbx5*, and* Myocd* downregulation in a specific microRNA-upregulation context in response to tachycardia-induced AF. From this perspective, our data would suggest that higher levels of miR-21, miR-10b, and miR-1 in the LA myocardium contribute to arrhythmogenesis via perturbation of* Pitx2*,* Tbx5*, and* Myocd* signaling pathways, respectively.

## 5. Conclusions

In conclusion, we herein demonstrate that even short-lasting atrial tachyarrhythmia is associated with significant miRNA remodeling coupled with decreased expression of TFs with potential roles in arrhythmogenesis. We propose that the downregulation of* Pitx2c, Tbx5*, and* Myocd* may be permissive for future AF development. This study provides, therefore, previously unknown insights into interrogating miRNA-TF regulations underlying paroxysmal AF development in a large animal model, with potentially important implications from a biomedical perspective.

## Figures and Tables

**Table 1:** The miRNAs significantly up- or downregulated in the paced LA, ordered by the fold change (FC).

miRNA	FC	Direction	*p* value	Reported expression in^∗^	Reference
ssc-miR-7140-3p	3,92	Up	0,0199	Porcine lung	[25]
ssc-miR-4335	3,22	Up	0,0540	Porcine intestine	[26]
ssc-miR-129b	2,90	Up	0,0520	Catfish heart	[27]
ssc-miR-296-5p	2,35	Up	0,0530	Human placenta	[28]
ssc-miR-4334-5p	2,22	Up	0,0072	Porcine intestine	[26]
ssc-miR-4339	2,16	Up	0,0499	Porcine intestine	[26]
ssc-miR-182	2,13	Up	0,0021	Human skeletal muscles	[29]
ssc-miR-4332	1,95	Up	0,0339	Porcine intestine	[26]
ssc-miR-1249	1,90	Up	0,0550	Rat pancreas	[30]
ssc-miR-1307	1,83	Up	0,0542	Human blood cells	[31]
**ssc-miR-671-3p**	1,63	Up	0,0492	Human atria	[23]
ssc-miR-423-3p	1,61	Up	0,0025	Rat hippocampus	[32]
**ssc-miR-23b**	−1,10	Down	0,0146	Human atria, AF	[20]
**ssc-miR-22-3p**	−1,19	Down	0,0181	Human atria, AF	[20]
**ssc-miR-143-3p**	−1,26	Down	0,0402	Human atria, AF	[20]
ssc-miR-324	−1,30	Down	0,0050	Human blood cells	[33]
ssc-miR-455-5p	−1,42	Down	0,0551	Human meningeal arteries	[34]
ssc-miR-326	−1,46	Down	0,0546	Human brain tissues	[35]
ssc-miR-362	−1,57	Down	0,0069	Human skeletal muscles	[36]
**ssc-miR-363**	−1,62	Down	0,0069	Human atria, AF	[21]
**ssc-miR-18b**	−1,67	Down	0,0550	Human atria, AF	[21]
ssc-miR-17-3p	−1,68	Down	0,0336	Mouse heart	[37]
**ssc-miR-24-2-5p**	−1,79	Down	0,0187	Human atria, AF	[23]
**ssc-miR-542-5p**	−1,85	Down	0,0318	Human atria, AF	[23]
ssc-miR-486	−1,88	Down	0,0365	Mouse skeletal muscles	[38]
**ssc-miR-27b-5p**	−1,96	Down	0,0509	Human atria, AF	[23]
ssc-miR-7	−2,37	Down	0,0211	Porcine heart	[39]
ssc-miR-30c-3p	−2,55	Down	0,0345	Porcine heart	[39]
ssc-miR-381	−2,69	Down	0,0540	Human skeletal muscles	[40]

^∗^Except the expression in malignant tumors and model cell lines. Bold, detected in AF patient samples.
